# Narcissism in patients admitted to psychiatric acute wards: its relation to violence, suicidality and other psychopathology

**DOI:** 10.1186/1471-244X-8-13

**Published:** 2008-02-27

**Authors:** Marit F Svindseth, Jim Aage Nøttestad, Juliska Wallin, John Olav Roaldset, Alv A Dahl

**Affiliations:** 1 Department of Psychiatry, Sunnmore Hospital, N-6026 Aalesund, Norway; 2 National University of Science and Technology, N-7440 Trondheim, Norway; 3 Department of Forensic Psychiatry, Broset, St. Olav's Hospital, N-7440 Trondheim, Norway; 4 Department of Social Sciences, Mälardalen University, S-635 13 Eskilstuna, Sweden; 5 Department of Clinical Cancer Research, The Norwegian Radiumhospital, Rikshospitalet University Hospital, N-0310 Oslo, Norway; 6 Faculty Division The Norwegian Radiumhospital, University of Oslo, N-0316 Oslo, Norway

## Abstract

**Background:**

The objective was to examine various aspects of narcissism in patients admitted to acute psychiatric wards and to compare their level of narcissism to that of an age- and gender-matched sample from the general population (NORM).

**Methods:**

This cross-sectional study interviewed 186 eligible acute psychiatric patients with the Brief Psychiatric Rating Scale (BPRS) and the Global Assessment of Functioning (GAF). The patients filled in the Narcissistic Personality Inventory-21 item version (NPI-21), The Hospital Anxiety and Depression Scale (HADS) and the Rosenberg Self-Esteem Scale. High and low narcissism was defined by the median of the total NPI-21 score. An age- and gender-matched control sample from the general population also scored the NPI-21 (NORM).

**Results:**

Being male, involuntary admitted, having diagnosis of schizophrenia, higher self-esteem, and severe violence were significantly associated with high narcissism, and so were also low levels of suicidality, depression, anxiety and GAF scores. Severe violence and high self-esteem were significantly associated with high narcissism in multivariable analyses. The NPI-21 and its subscales showed test-retest correlations ≥0.83, while the BPRS and the HADS showed lower correlations, confirming the trait character of the NPI-21. Depression and suicidality were negatively associated with the NPI-21 total score and all its subscales, while positive association was observed with grandiosity. No significant differences were observed between patients and NORM on the NPI-21 total score or any of the NPI subscales.

**Conclusion:**

Narcissism in the psychiatric patients was significantly associated with violence, suicidality and other symptoms relevant for management and treatment planning. Due to its trait character, use of the NPI-21 in acute psychiatric patients can give important clinical information. The similar level of narcissism found in patients and NORM is in need of further examination.

## Background

Narcissism describes the personality trait of an exceptional interest in and admiration for oneself [1]. The Narcissistic Personality Inventory(NPI) was developed to measure narcissism in the general population [2], and the 40 item version (NPI-40) has become the most commonly used self-rating scale for that purpose. Psychometric testing of the NPI-40 has mostly been done in undergraduate samples, and the scale has generally shown good internal consistency and test-retest reliability. Factor analyses have shown various solutions with four to seven factors [3-6].

The first data from the general population on the NPI 40 was published from Sweden by Kansi [7]. According to her findings, a revised version of the NPI with 29 items (NPI-29) was developed and factor analysis identified four separate factors. We examined the NPI-29 in a Norwegian population sample using structural equation modelling, and found that a 21-items version (NPI-21) showed better fit and similar external validity as the NPI-29 in the sample of acute psychiatric patients used in the present study [Svindseth M, Sørebø Ø, Wallin J, Nøttestad JA, Roaldset JO, Dahl AA. Structural equation modelling examination of and normative data for The Narcissistic Personality Inventory 29 item version (In preparation)]. The NPI-21 consists of the same four factors as the NPI-29 but with fewer items per factor: factor 1: Leadership/Power (5 items); factor 2: Exhibitionism/Self-admiration (6 items); factor 3: Superiority/Arrogance (5 items) and factor 4: Uniqueness/Entitlement (5 items).

Although narcissism has been a major focus for psychoanalysis and psychodynamic psychotherapy for a long time, and the narcissistic personality disorder was introduced by DSM-III in 1980, limited research has been done on narcissism in ordinary psychiatric patients. The reason may be that narcissism is not covered by the common lists of mental symptoms [8]. However, narcissism has been associated with aggression, violence, depression, and suicidality, symptoms and behaviours that are of major concern to psychiatry and society [9-11]. Search in databases retrieved only one empirical paper on narcissism in hospitalized psychiatric patients. Prifitera & Ryan [12] administered the NPI-40 to 50 such patients and reported significant correlations between the NPI score and the scores on several other personality trait scales. Our search did neither find any publications on narcissism in psychiatric patients compared to normative data on narcissism in the general population.

Thus the aims of this study of narcissism in patients admitted to an acute psychiatric service were fourfold: 1) To explore the characteristics of patients with high and low narcissism scores; 2) To explore the trait versus state character of the NPI-21; 3) To explore the associations between narcissism and various mental symptoms; and 4) To compare the narcissism scores in the patient sample to those of an age and gender adjusted sample from the general population (NORM).

## Methods

### Setting

The Aalesund Hospital is located in the city of Aalesund at the North-western coast of Norway. The psychiatric ward has four acute units. Two closed (8 beds each) and two open wards (one with 8 beds and one with 10 beds), all with separate patient rooms. The hospital serves a geographical sector of about 95.000 people ≥18 years of age.

### Patient sampling

Consecutively admitted patients to the two closed acute units during the period between March 1^st^, 2005 and October 15^th^, 2006, were invited to the study if they were eligible. Exclusion criteria were dementia or organically based confusion, manic or hypomanic states, re-admittance during the sampling period, poor ability to speak Norwegian, or discharge within 48 hours.

The study included both involuntary and voluntary admitted eligible patients. All involuntary patients were invited to the study. Due to a majority of voluntary patients, only those admitted on specifically defined days of the week were invited. All patients had a project interview within three days after admission, except a minority who were interviewed within the first week due to the severity of their mental state.

During the sampling period 191 patients with involuntary status were admitted, and 54 were re-admissions, 78 did not meet the eligibility criteria, 8 declined to take part or withdrew their consent, and 7 were lost due to administrative reason. This left 98 involuntary patients for the study. On the defined days, 160 voluntary patients were admitted, 48 did not meet the eligibility criteria, 13 declined to take part or withdrew their consent, and 11 were lost due to administrative reason. This left 88 voluntary patients for the study. The total sample of this study thus consisted of 186 patient. Among them 147 patients (79%) were re-interviewed within 24 hours prior to their discharge.

### Measurements

#### The Narcissistic Personality Inventory (NPI)

We used the NPI-21 developed by our group, derived from the NPI-29 (Table 1). The NPI-21 consists of 21 dual statements among which one is considered indicative of narcissism. Each statement is scored 'yes' or 'no', and there is no time limit as to the evaluation. Based on summation of the relevant items, the total NPI score as well as the four factor scores are calculated.

**Table 1:** The NPI-21 items according to factors.

*Factor 1. Leadership/Power*
1. I have a natural talent for influencing people
8. I will be a success
10. I see myself as a good leader
11. I am assertive
33. I would prefer to be a leader
*Eliminated from NPI-29: items #5, #17 and #27*

*Factor 2. Exhibitionism/Self-admiration*

4. I know I am good because everybody keeps telling me so
15. I like to display my body
19. I like to look at my body
20. I am apt to show off if I get the chance
26. I like to be complimented
29. I like to look at myself in the mirror
*Eliminated from NPI-29: item #38*

*Factor 3. Superiority/Arrogance*

16. I can read people like a book
21. I always know what I am doing
22. I rarely depend on anyone else to get things done
31. I can live my life in any way I want to
35. I can make anybody believe anything
*Eliminated from NPI-29: item #39*

*Factor 4. Uniqueness/Entitlement*

2. Modesty does not become me 9. I am an extraordinary person
18. I want to amount to something in the eyes of the world
34. I am going to be a great person
36. I am born a leader
*Eliminated from NPI-29: items # 14 25, 28*

# All items numbers according to the NPI-40.

In order to study the low and high narcissism, the sample was separated into two groups based on the median of the total NPI-21 score on admission: low narcissism (total NPI-21 score <5), and high narcissism (total NPI-21 score ≥ 5), respectively. The internal consistency of the total NPI-21 in the patient sample (with the NORM values within a parenthesis) was for the NPI-21 total α = 0.83 (α = 0.75), factor 1 α = 0.63 (α = 0.73), factor 2 α = 0.67 (α = 0.64), factor 3 α = 0.52 (α = 0.43) and factor 4 α = 0.61 (α = 0.52).

*The Brief Psychiatric Rating Scale (BPRS) *is a clinician-rated test designed to assess status of and changes in severity of psychopathology [13,14] with focus on symptoms that are common in patients with psychotic disorders. The instrument includes 24 items of psychopathology, and the time-frame of evaluation is the day of the interview. Items are rated on a 7-point Likert scale anchored from 1 (not present) to 7 (extremely severe), and thus higher scores represent more psychopathology. We used a version of BPRS with explanations of each of the rating points.

Eight experienced registered psychiatric nurses who had been trained by the first author, did the patient interviews and assisted the patients in filling in the self-report forms if necessary. Training of the nurses involved study of written material on the BPRS, taking part in group-discussions and making three patient interviews supervised by the first author. Reliability testing of the eight interviewers showed correlation coefficients of 0.87 – 0.97 compared to those of the supervisor and between the interviewers of 0.74–0.97 based on the BPRS scorings of three patients.

Various factor analytic studies have identified several subscales of the BPRS that have descriptive utility, and we used five of them: Thinking disturbance (α = 0.61 in our patient sample), Withdrawal/retardation (α = 0.58), Hostility/suspiciousness (α = 0.61), Anxiety/depression (α = 0.57), and Activation (α = 0.61). *Suicidality *was defined as a score of ≥score 4 ("Moderate") on the BPRS item #4 during the admission project interview or equivalent severity documented in the medical records. We also made special analyses of the BPRS item #3 Depression and item #8 Grandiosity due to their significant relationship to narcissism.

*The Hospital Anxiety and Depression Scale (HADS) *is a self-rating scale consisting of seven items measuring anxiety (HADS-A) and seven items measuring depression (HADS-D) during the last week [15]. The HADS-D focuses mainly on reduced ability to feel pleasure (anhedonia), and the HADS-A on generalized anxiety relating to worries and fear of what might happen in the future. Each item has scores from 0 (minimum presence) to 3 (maximum presence). The internal consistencies of the HADS-A and the HADS-D in the patient sample were α = 0.85 and α = 0.82, respectively. The correlation between HADS-D and BPRS-depression (item #3) was r = .39 and between HADS-A and BPRS anxiety (item #2) r = .51 at admission.

*The Rosenberg Self-Esteem Scale (RSES) *originally consisted of 10 statements scored on a four-point scale range from "strongly agree" to "strongly disagree" considering "your *general *feelings about yourself" (no time frame) [16]. In this study we included the four items from the RSES used in another Norwegian study [17]. The range of the RSES sum score was from 0 to 12, with higher score meaning better self-esteem. The internal consistency of the four-item RSES version in our patient sample was α = 0.85.

*Scale for the Prediction of Aggression and Dangerousness *has been modified in Norwegian studies [18]. We recorded the violence from the first contact leading to admission to discharge through both observations in the wards and documentation in the medical records. We classified violence according to the Intensity subscale into: "No violence" "Threats", "Mild violence", "Moderate violence" and "Severe violence". We recoded this variable into three categories: No violence, mild/moderate (including threats) and severe violence.

### Information from medical records

The psychiatrists' documentation of violence and suicidality was used in the rating of these symptoms.

*ICD-10 diagnoses *were given by a psychiatrist according to ICD-10 manual [19] at the end of the index hospitalization. Only the main diagnosis was used in this study.

*Global Assessment of Functioning (GAF) *is an observer-based rating scale for the current overall functioning of a patient on a continuum from the most severe mental disorder to complete mental health that was defined as Axis V of the DSM-IV. Scale values range from 1 (sickest individual) to 100 (the healthiest individual). The GAF is regarded as a reliable instrument [20]. The inter-rater reliability of GAF has been found to be ≥0.61, and concurrent validity has been found satisfying [21]. A recent study from Norway examined the reliability and precision of the GAF, split into functions (GAF-F) and symptoms (GAF-S) [22]. Both function and symptom scales were found to be highly generalizable (correlation score between symptom and function score were r = 0.61). The GAF-F and GAF-S were scored by the psychiatrist at the intake interview.

#### Demographic variables

Level of education was divided into three classes (≤9, 10–11, ≥12 years) based on completed school years; income status was dichotomized (paid work or self-employed, versus unemployed or pensioned). Civil status was divided into paired (married, cohabiting) and non-paired relationships.

### Normative sample (NORM)

A random sample of 750 men and 750 women, with the same age and gender distribution as the general Norwegian population between 20 and 79 years, was drawn from official mailing lists. They got an invitation to fill in the NPI-29 and basic demographic variables anonymously. We got 407 valid answers (27% response rate). Based on 5 years age intervals, we drew one age- and gender matched control for each of the 186 patients of the study.

### Statistical analyses

Continuous measures were analyzed by independent sample t-tests or analysis of variance with adjustment for gender. Skewed distributions were examined with non-parametric tests as appropriate. Categorical variables were examined with the χ^2 ^test. Effect sizes (ES) were calculated on the significant dimensional differences and 2 × 2 contingency tables between the groups according to Cohen's coefficient d, and d values ≥0.40 were considered as clinically significant [23,24]. Partial correlations controlled for gender, between the BPRS items and the NPI-21 were examined with Pearson's correlation coefficients. Internal consistencies of scales and subscales were examined with Cronbach's coefficient α. The associations between relevant independent variables and high narcissism (dependent variable with low narcissism as reference) in the patient sample were examined with logistic regression analyses. The strength of associations was expressed as odds ratios (ORs) with 95% confidence intervals (95%CI).

The data were analyzed on SPSS for PC version 13.0. Due to multiple comparisons the significance level was set at p < 0.01, and all tests were two-tailed.

### Ethics

The study was approved by the Regional Committee of Ethics in Medical Research of Mid-Norway, and The Norwegian Data Inspectorate. All patients gave written informed consent after full oral and written information.

## Results

### Sample characteristics

In the total sample (N = 186) 41% were females and 59% were males. The mean age was 37.3 (SD 13.4) years, and 27% were married or cohabiting, and the same proportion was working. Concerning type of admissions 53% were involuntary and 47% was voluntary (Table 2).

**Table 2:** Characteristics of the patient sample.

**Variables**	**Low narcissism **(n = 88)	**High narcissism **(n = 98)	**P**	**Effect Size**	**Total sample **(n = 186)
*Age*, mean (SD)	39.2 (13.5)	35.7 (13.1)	0.07		37.3 (13.4)

	**N (%)**	**N (%)**			

*Gender*			<0.001	0.53.	
Females	48 (55)	28 (29)			76 (41)
Males	40 (45)	70 (71)			110 (59)
*Civil status*			0.44		
Married/Cohabiting	26 (29)	24 (24)			50 (27)
Single	62 (71)	74 (76)			136 (73)
*Level of education*			0.22		
≤ 9 years	34 (39)	27 (28)			61 (33)
10 – 11 years	31 (35)	45 (46)			76 (41)
≥ 12 years	23 (26)	26 (26)			49 (26)
*Job status*			0.83		
Working	23 (26)	27 (28)			50 (27)
Unemployed, pensioned	65 (74)	71 (72)			136 (73)
*Admission status*			0.001	0.49	
Voluntary	53 (60)	35 (36)			88 (47)
Involuntary	35 (40)	63 (64)			98 (53)
*Diagnosis*					
Substance abuse	5 (17)	25 (26)	0.002		40 (21)
Schizophrenia	14 (16)	34 (35)			48 (26)
Major depressions	30 (34)	24 (24)			54 (29)
Neurotic disorders	19 (22)	7 (7)			26 (14)
Personality disorders	10 (11)	8 (8)			18 (10)
*Violence*			<0.001		
None	58 (66)	42 (43)			100 (54)
Mild/moderate	28 (32)	37 (38)			65 (35)
Severe	2 (2)	19 (19)			21 (11)
*Suicidal on admission*			<0.001	0.58	
No	33 (37)	65 (66)			98 (53)
Yes	55 (63)	33 (34)			88 (47)

	***Mean (SD)***	***Mean (SD)***			

BPRS*					
Total score	54.9 (14.3	55.3 (14.8)	0.78		55.1 (14.5)
Thinking disturbance	6.4 (3.9)	7.1 (3.9)	0.26		6.8 (3.9)
Withdrawal/retardation	4.5 (2.5)	3.5 (1.8)	0.003	0.47	3.9 (2.2)
Anxiety/Depression	11.1 (3.8)	8.7 (3.9)	0.001	0.62	9.8 (4.0)
Hostility/suspiciousness	5.9 (3.4)	7.2 (3.9)	0.08		6.6 (3.8)
Activation	6.4 (3.6)	7.5 (3.7)	0.11		7.0 (3.7)
Suicidality	4.0 (2.3)	2.5 (2.0)	<0.001	0.70	3.2 (2.2)
Rosenberg self-esteem*	4.2 (2.6)	7.5 (3.0)	<0.001	1.17	6.0 (3.2)
HADS*					
Depression	11.0 (4.0)	7.3 (4.5)	<0.001	0.87	9.0 (4.7)
Anxiety	13.5 (4.1)	10.6 (5.4)	<0.001	0.60	12.0 (5.0)
GAF-Function*	42.9 (9.7)	40.0 (11.5)	0.08		41.4 (10.7)
GAF-Symptoms*	41.3 (10.9)	37.8 (11.1)	0.04		39.5 (11.1)

*ANOVA with adjustment for gender ^¤ ^Cohen's d is the between group effect size

The total sample was separated into patients with low (N = 88) and high narcissism score (N = 98) based on the median of the total NPI-21 total score on admission. The high narcissism group contained more males, had higher proportion of involuntary status, more patients with schizophrenia, fewer with major depressive disorders, and they also had more frequently episodes with severe violence, and were less frequently suicidal at admission (Table 2). The difference concerning gender, admission status and suicidality at admission were all clinically significant.

Since the numbers of patients who declined or were lost were so few, no attrition analyses were performed.

### Comparison of patients with high and low narcissism

The BPRS subscale scores on Withdrawal/Retardation, Anxiety/Depression and Suicidality were all significantly lower in the high narcissism group (ES 0.47, 0.62 and 0.70 respectively), while no significant differences were observed as to BPRS Total score, Thinking disturbance, Hostility/suspiciousness or Activation (Table 2).

HADS-depression and the HADS-Anxiety mean scores were significantly lower in the high narcissism group, while the RSES mean score was significantly higher in that group (Table 2). The effect sizes of HADS-Anxiety, HADS-Depression and RSES were all clinically significant (ES ≥0.60).

### Temporal stability of measures

The NPI-21, the BPRS, the HADS and the RSES tests were performed at admission and discharge. Among the 186 patients included at admission, 147 (79%) also had ratings at departure after a mean time of 20 days (range 2–197 days). The test-retest correlations were r = 0.94 for NPI-21 total score, r = 0.93 for Factor 1, r = 0.92 for Factor 2, r = 0.83 for Factor 3 and r = 0.89 for Factor 4 (Table 3). The differences between the means at admission and discharge all showed effect sizes ≤0.15.

**Table 3:** Changes of scores from admission to discharge (N = 147)*.

**Variables**	**Ratings at Admission **(n = 147)	**Ratings at Discharge **(n = 147)	**P.**	**Effect size**^¤^	**r**
	***Mean (SD)***	***Mean (SD)***			

*NPI-21*					
Total score	5.3 (4.2)	4.7 (3.6)	<0.001	0.15	0.94
Leadership, factor 1	1.5 (1.5)	1.4 (1.5)	0.006	0.09	0.93
Exhibitionism, factor 2	1.4 (1.6)	1.2 (1.4)	0.02	0.08	0.92
Superiority, factor 3	1.5 (1.4)	1.4 (1.3)	0.01	0.13	0.83
Entitlement, factor 4	0.9 (1.2)	0.8 (1.1)	0.02	0.12	0.89
*BPRS*					
Total score	53.8 (14.4)	35.8 (8.6)	<0.001	1.52	0.60
Thinking disturbance	6.5 (3.8)	6.1 (3.1)	0.24	0.10	0.52
Withdrawal/retardation	3.9 (2.2)	5.7 (2.2)	<0.001	0.83	0.61
Anxiety/Depression	9.9 (4.1)	8.0 (2.8)	<0.001	0.55	0.74
Hostility/suspiciousness	6.3 (3.7)	4.0 (1.7)	<0.001	0.81	0.49
Activation	6.5 (3.8)	4.2 (1.3)	<0.001	0.79	0.41
Depression (item #3)	3.6 (1.9)	2.2 (1.8)	<0.001	0.76	0.69
Suicidality (item #4)	3.4 (2.2)	1.6 (1.0)	<0.001	0.99	0.63
Grandiosity (items #8)	1.9 (1.7)	1.6 (1.3)	0.002	0.20	0.74

Rosenberg self-esteem	6.4 (3.2)	5.1 (2.6)	<0.001	0.43	0.82

HADS-Depression	9.2 (4.6)	6.5 (3.5)	<0.001	0.67	0.79
HADS-Anxiety	12.0 (5.1)	8.4 (3.9)	<0.001	0.80	0.75

*Two-related samples test ^¤ ^Cohen's d is the between group effect size

In contrast, HADS-A showed a correlation of 0.75 and an effects size of 0.80, and the corresponding findings for HADS-D were 0.79 and 0.67, respectively. For the BPRS total mean score and subscale mean scores the correlations ranged between 0.41 and 0.74, and except for the Thinking disturbance subscale, the effect sizes were all ≥0.55. For the BPRS items of depression, suicidality and grandiosity the correlations ranged from 0.63 to 0.74. However, grandiosity only showed a small effect size of change (0.20) while the values for depression and suicidality were >0.75.

We have also tested the correlations of NPI-21 total score at admission and discharge for the ICD-10 diagnostic groups and found that for substance abuse r 0.97, schizophrenia r 0.92, major depressive disorders r 0.94, neurotic disorders r 0.83 and personality disorders r 0.97.

### Partial correlations of the NPI-21 versus the BPRS and other measures

Partial correlation analyses of narcissism were performed on the scores at admission in the total sample, and were adjusted for the significant gender difference of narcissism. The NPI-21 total score was significantly correlated with 13 of the 24 BPRS items, while NPI-21 factor 2 and 4 were correlated with 11 and 12 BPRS items, respectively. In contrast, NPI-21 factor 1 was associated with 6 and factor 3 with 4 BPRS items only (Table 4). The BPRS items of Depression and Suicidality showed significant, negative association with the NPI-21 total score and all the factor scores. The BPRS item of Grandiosity showed significant and positive association with the NPI-21 total scores and all the factor scores. Eight BPRS items: Somatic concern, Anxiety, Hallucinations, Self-neglect, Disorientation, Emotional withdrawal, Tension and Distractability showed no significant associations with any of the NPI-21 scores. The other BPRS items were significantly associated with one or more of the NPI-21 scores.

**Table 4:** Partial correlation matrix for the BPRS, the HADS, the Rosenberg self-esteem, the GAF, and violence and suicidality and the NPI-21 total and NPI-21 factors measured at admission and controlled for sex.

**BPRS items**	**NPI Total**	**NPI Factor 1**	**NPI Factor 2**	**NPI Factor 3**	**NPI-21 Factor 4**
1. Somatic concern	-0.06	0.01	-0.07	-0.05	-0.06
2. Anxiety	-0.09	-0.03	-0.10	-0.08	-0.07
**3. Depression**	-0.45*	**-0.35**	**-0.36**	**-0.25**	**-0.41**
**4. Suicidality**	**-0.42**	**-0.38**	**-0.33**	**-0.28**	**-0.27**
**5. Guilt**	**-0.24**	-0.18	**-0.25**	-0.07	**-0.22**
**6. Hostility**	**0.20**	0.11	**0.21**	0.08	**0.20**
**7. Elated mood**	**0.28**	0.18	**0.30**	0.16	**0.20**
**8. Grandiosity**	**0.42**	**0.25**	**0.31**	**0.34**	**0.40**
**9. Suspiciousness**	0.18	0.12	0.10	0.12	**0.25**
10. Hallucinations	0.06	0.08	-0.02	0.06	0.06
**11. Unusual thoughts**	**0.27**	0.18	0.18	0.15	**0.36**
**12. Bizarre behavior**	**0.22**	0.15	**0.25**	0.05	**0.20**
13. Self-neglect	-0.04	-0.10	0.04	-0.12	0.08
14. Disorientation	0.09	0.04	0.11	0.03	0.12
**15. Conceptual disorganisation**	**0.19**	0.14	0.17	0.13	0.13
**16. Blunted affect**	-0.17	**-0.20**	**-0.21**	-0.03	-0.03
17. Emotional withdrawal	-0.02	-0.04	-0.07	-0.03	0.12
**18. Motor retardation**	**-0.26**	**-0.23**	**-0.27**	-0.12	-0.16
19. Tension	0.01	0.01	-0.02	-0.03	0.07
**20. Uncooperativeness**	**0.22**	0.12	0.14	**0.20**	**0.23**
**21. Excitement**	**0.27**	0.17	**0.31**	0.11	**0.20**
**22. Distractability**	0.14	0.08	0.16	0.08	0.09
**23. Motor hyperactivity**	**0.26**	**0.23**	**0.24**	0.15	0.19*
**24. Mannerism and posturing**	0.17	0.11	0.13	0.08	**0.24**
*No of significant correlations*	*13*	*6*	*11*	*4*	*12*
***Other measures***					
**HADS-Anxiety**	**-0.23**	**-0.22**	-0.17	**-0.22**	-0.05
**HADS-Depression**	**-0.38**	**-0.34**	**-0.35**	**-0.22**	-0.18
**Rosenberg Self-esteem scale**	**0.53**	**0.47**	**0.43**	**0.36**	**0.31**
**GAF-Symptoms**	**-0.30**	**-0.19**	**-0.30**	-0.16	**-0.25**
**GAF-Function**	**-0.23**	-0.18	-0.19	-0.12	**-0.21**
**Violence**	**0.32**	0.16	**0.35**	0.16	**0.28**
**Suicidal on admission**	**-0.41**	**-0.38**	**-0.31**	**-0.26**	**-0.27**

*Boldfaced values: p < 0.01.

The RSES was positively, and suicidality on admission negatively, associated with all the NPI-21 measures. The HADS-Depression, the HADS-Anxiety and the GAF-Symptom score were significantly negatively associated with the NPI-21 total score and three of the factor scores. The GAF-Function was negatively associated with the NPI-21 total score and factor 4, while factors 2 and 4 were positively associated with violence (Table 4).

In order to explore the stability of the observed correlations, we also checked the partial correlations of the NPI-21 and its subscales with the BPRS items of depression, suicidality and grandiosity at discharge (N = 147). The significant correlations between these items and the NPI-21 total score were confirmed, while the correlations with the subscale scores were more variable (data not shown).

### Variables significantly associated with high narcissism

Univariate analyses showed that being male, involuntary admission, severe violence, higher scores on RSES and having a diagnosis of schizophrenia were significantly associated with high narcissism. The BPRS subscales of Withdrawal/Retardation, Anxiety/Depression and Suicidality as well as HADS-Depression and HADS-Anxiety were significantly associated with low narcissism (Table 5). When these variables were entered into a multivariable model severe violence and higher RSES scores were significantly associated with high narcissism (Table 5).

**Table 5:** Univariate and multivariate logistic regression analyses of selected variables and presence of high narcissism (low narcissism = reference).

**Variables**	**Univariate**			**Multivariate**		
	**OR**	**95%CI**	**P**	**OR**	**95% CI**	**P**

Age	0.98	0.96 – 1.00	0.08			
Being male	3.00	1.64 – 5.50	<0.001	1.92	0.90–4.11	0.09
Involuntary admission	2.73	1.51 – 4.94	0.001	1.20	0.50 – 2.87	0.69
*Violence*						
No violence (ref.)	1.00			1.00		
Mild/moderate	1.83	0.97 – 3.43	0.06	1.21	0.51 – 2.87	0.67
Severe	13.12	2.90 – 59.40	0.001	11.46	2.02–65.60	0.006
*BPRS subscales/item*						
Anxiety/Depression	0.86	0.79 – 0.93	<0.001	1.09	0.90 – 1.23	0.18
Withdrawal/Retardation	0.81	0.70 – 0.93	0.003	0.83	0.68 – 1.00	0.05
Hostility/Suspicion	1.10	1.01 – 1.19	0.03			
Suicidality	0.73	0.64 – 0.84	<0.001	0.86	0.70 – 1.05	0.14
Rosenberg Self-esteem	1.50	1.31 – 1.71	<0.001	1.37	1.15 – 1.62	<0.001
HADS-Depression	0.82	0.76 – 0.89	<0.001	0.95	0.85 – 1.06	0.35
HADS-Anxiety	0.88	0.83 – 0.94	<0.001	0.88	0.83 – 0.94	0.07
GAF-Symptoms	0.97	0.95 – 0.99	0.03			
Nonschizophrenia (ref.)	1.00					
Schizophrenia	2.81	1.39 – 5.69	0.004	1.06	0.37 – 3.04	0.92

### Comparison of patients and the NORM

**Figure 1:**
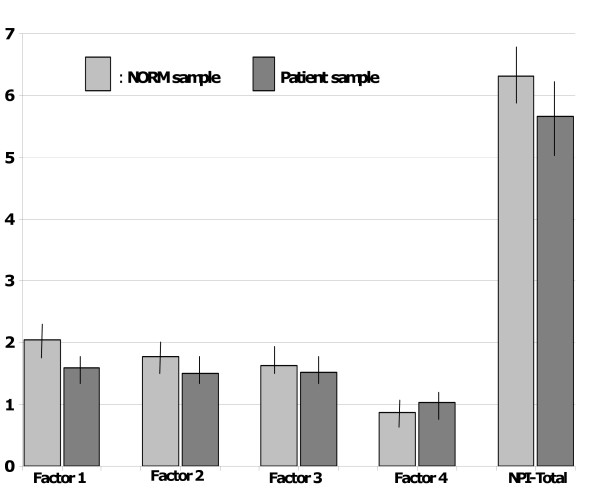
Mean scores with 95% confidence intervals of NPI-21 total and subscales in the patient sample and the NORM sample.

The NORM sample was matched with the patient sample on age and gender. Fewer patients were in paired relations (27% versus 78%, p < 0.001, ES = 1.08), were employed (27% versus 82%, p < 0.001, ES = 1.18), or held higher level of education (≥13 completed school years) (26% versus 51%, p < 0.001, ES = 0.52). In ANOVA analyses adjusted for paired relation, level of education and employment, no significant differences were observed between patients and NORM concerning the NPI-21 total score, nor any of the NPI-21 factor scores (Figure 1).

## Discussion

### Main findings

In this sample of acute psychiatric patients we observed that male gender, involuntary admission, severe violence, and high self-esteem were significantly associated with a high level of narcissism. The level of suicidality, withdrawal/retardation as well as anxiety and depression were significantly associated with low level of narcissism. In test-retest examination from admission to discharge the NPI-21 total and subscale scores showed considerable higher correlation coefficient and lower effects sizes than the BPRS total and subscale score and the HADS-A and HADS-D scores, thus supporting the NPI-21 as a trait measure. On admission narcissism as measured by the NPI-21 and its subscales had a significant positive correlations with BPRS grandiosity, and a significant negative correlation with BPRS depression and suicidality, but at discharge these relations only held up for the NPI-21 total score and to an variable extent for the subscale scores. On admission eight BPRS symptoms had no significant associations with narcissism at all, while 13 BPRS symptoms showed one or more significant associations with the NPI-21 total or its subscales. In multivariable analyses high narcissism in the patients were significantly associated with severe violence and higher self-esteem. Finally, no significant differences were observed in the NPI-21 total or subscale mean scores between the patients and an age and gender-matched sample from the general population.

### Stability of the NPI-21 ratings

A fundamental premise for this study was that the NPI-21 measures a trait characteristic. The test-retest correlations from admission to discharge of the NPI-21 and its subscales were higher than those observed for the BPRS total and its subscales (except for Thinking disturbance), the BPRS items of depression and suicidality and the HADS-A and the HADS-D.

The effect sizes of the differences between admission and discharge were minimal for the NPI-21 total and its subscales (≤0.15), while they were considerable for the BPRS subscales and the depression and suicidality items and the HADS (≥0.55). In contrast, the effect size for grandiosity was 0.20. Finally, no significant differences were observed between the mean NPI-21 total and subscales scores of patients at admission and normative data from the general population.

We consider that this evidence taken together supports the consideration of the NPI-21 as a valid trait measure of narcissism in the patient sample. On the other hand, some of our findings indicate instability of the narcissism scores and further study of this instability is necessary before narcissism can be used in a general population sample to predict violence for example.

### Comparisons with other studies of narcissism

This is the first empirical study of narcissism in a sample of patients admitted to an acute psychiatric ward reporting more extensive findings, since Prifitera & Ryan [12] only reported significant correlations between the NPI score and other personality traits. Since there have been no other systematic empirical studies of narcissism in patients admitted to acute psychiatric wards, we have to relate our results to those of various clinical studies of narcissism. The higher prevalence of severe violence in patients with higher narcissism supports the statement by Nestor [25] that narcissistic traits are associated with risk of violence. In his study patients with high levels of narcissism felt entitled to react aggressively upon what they sensed as threats or humiliations. Aggression could thus function as a defence against hurt of a vulnerable self, but could also restore self-esteem after humiliations.

The most relevant predictors for violence are probably threatened egotism and inflated or unstable self-esteem [9,26]. Our results confirm the associations between violence and high narcissism. Even if our sample had a low number of participants who showed severe violence, the results are compatible with theories that link violence to narcissism [27]. However, we did not collect other data than violence concerning antisocial behaviour, so that we are not able to tell the proportion of patients that could be considered as psychopaths.

Simon [28] found that sudden threats to an individual's self-esteem may increase the level of grandiosity in order to regain its sense of stable self-esteem. It is likely to assume that psychiatric admissions, especially involuntary ones could be experienced as threatening to the ego, and that the individuals try to compensate with inflated self-esteem [10]. This is supported by the significant associations between the NPI-21 measures and the BPRS Grandiosity item.

Patients with high narcissism scored significantly lower on suicidality and depression at admission than those with low narcissism. The level of narcissism should therefore be considered in conjunction with other factors that are associated with increased risk of suicide, like depression, substance dependence and deliberate self-harm.

The finding that high narcissism is strongly associated with high self-esteem is in agreement with other findings [10]. Grandiosity is a symptom of high self-esteem, and positively associated with the NPI-21 total score as well as all the factor scores. However we also found that the BPRS grandiosity item is a trait characteristic, and this finding could be considered as support to the content validity of narcissism.

### New findings

To our knowledge the correlations between the BPRS (state measure) items and the NPI-21 total and subscale scores (trait measure) have not been examined before in acute psychiatric patients. We found that the number of significant associations between the NPI-21 factors and the BPRS items varied considerably, which indicate that the relation between aspects of narcissism and various psychopathological symptoms could be unstable.

Among the clinically relevant findings are the negative correlations between narcissism and depression and suicidality. It is also of interest that self-rated depression showed the same significant correlations with narcissism as the clinician-rated depression. However, clinician-rated BPRS Anxiety did not show any significant correlations with narcissism, while the opposite was true for the self-rated HADS anxiety subscale. It is also a new finding that eight of the BPRS items showed no significant association with narcissism in these patients.

Another new finding is that the GAF Symptom and GAF Function scales which measure general mental functioning and its practical consequence consistently were negatively associated with the NPI-21 total score and the subscale scores. This implies that higher levels of narcissism are significantly associated with poorer of mental health and poorer function compared to lower levels of narcissism.

The higher narcissism score in involuntary patients could be explained by the experience of infringement, loss of personal control and of threat associated with that type of admissions. An alternative view would be that patients high in narcissism were less co-operative and more violent, and therefore involuntary admission had to be used more frequently.

The comparison of narcissism in patients and NORM also represents new findings. Surprisingly no significant differences were observed between these two groups. As mentioned before, this finding supports the evidence for the NPI-21 as a trait measure. An explanation of the lack of difference could be *response shift *which refers to the change in the meaning of one's evaluation of a construct as a result of a change in one's internal standards of measurement, a change in one's values, or a change in one's definition of the construct [29]. On all the demographic variables the patients are significantly worse off, but still their narcissism does not differ.

Not surprisingly, we observed that men scored significantly higher than women on the NPI-21. Both Reichman et al [30] and Foster et al [31] concluded that the expression of narcissism was different between the genders, as men often showed a greater sense of uniqueness and entitlement while women displayed less overt narcissistic characteristics.

### Strength and limitations

This study is, to our knowledge, the first to more fully characterize narcissism in a sample of acutely hospitalized psychiatric patients, and to compare their narcissism to that of matched controls from the general population. Our response rate is high among the patients, and 186 of 225 eligible respondents (83%) were included in the study. The NPI-21 version was derived from the NPI-29 of the Swedish population-based study, and further elaborated upon by structural equation modelling, which is considered a state of the art method for such investigations.

Our study also has some important limitations. The NPI-21 instrument used to measure narcissism has been derived from psychometric analyses that are under consideration for publication and external validity has to be confirmed. Both NPI-21 factor 3 and 4 showed low internal consistencies in both the patients and the controls.

We made many statistical comparisons in this study, which implies a risk for type I statistical errors. We reduced that risk by setting the p-value at <0.01. However, there still is a small risk of artificial significant associations.

A major limitation is the low participation rate in the NORM sample, which is a common problem in surveys of the general population on sensitive issues. Other studies have demonstrated only modest differences in prevalence estimates and socio-demographic distribution when comparing results by individuals responding after a reminder and initial responders [32,33]. The lack of exact knowledge about representativity of the normative sample is a problem in this study.

## Conclusion

We observed that the level of narcissism measured by the NPI-21 in patients admitted to acute psychiatric wards was significantly associated with major clinical features like violence, suicidality, depression, self-esteem and other psychopathology relevant for management and treatment planning. Since the findings supported the NPI-21 as a trait measure, these results imply that use of the NPI-21 for patients in the acute ward setting could give important information and be easily feasible.

## Competing interests

The author(s) declare that they have no competing interests.

## Authors' contributions

MFS contributed to acquisition of data, statistical analysis and interpretation of data, as well as drafting of the manuscript. JAN has helped to draft the manuscript and given comments on the paper. JW has been involved in interpretation of data and contributed with important comments on the paper. JOR supervised the collection of data and helped to draft the manuscript. AAD contributed to statistical analysis and interpretation of data, as well as manuscript design and coordination. All authors have contributed, read, and approved the final manuscript.

## Pre-publication history

The pre-publication history for this paper can be accessed here:


